# Reimagining eating disorder spaces: a qualitative study exploring Māori experiences of accessing treatment for eating disorders in Aotearoa New Zealand

**DOI:** 10.1186/s40337-023-00748-5

**Published:** 2023-02-15

**Authors:** Mau Te Rangimarie Clark, Jenni Manuel, Cameron Lacey, Suzanne Pitama, Ruth Cunningham, Jennifer Jordan

**Affiliations:** 1 Department of Māori Indigenous Health Innovation, University of Otago, PO Box 4345, Christchurch, New Zealand; 2 Department of Psychological Medicine, University of Otago, Christchurch, New Zealand; 3 Department of Public Health, University of Otago, Wellington, New Zealand; 4 Te Whatu Ora, Waitaha - Canterbury, Canterbury, New Zealand

**Keywords:** Eating disorders, Ethnic minority, Health disparity, Health service use, Indigenous, Māori

## Abstract

**Background:**

Health, illness, and the body are conceptualized within the cultural context of a society. The values and belief systems of a society, including media portrayals, shape how health and illness present. Traditionally, Western portrayals of eating disorders have been prioritized over and above Indigenous realities. This paper explores the lived experiences of Māori with eating disorders and their whānau (family/support system) to identify the enablers and barriers to accessing specialist services for eating disorders in New Zealand.

**Method:**

Kaupapa Māori research methodology was used to ensure the research supported Māori health advancement. Fifteen semi-structured interviews were completed with Māori participants including; those with an eating disorder diagnosis (anorexia nervosa, bulimia nervosa, and binge eating disorder), and/or their whānau. Structural, descriptive, and pattern coding was undertaken within the thematic analysis. Low’s spatializing culture framework was used to interpret the findings.

**Results:**

Two overarching themes identified systemic and social barriers to accessing treatment for Māori with eating disorders. The first theme, was space, that described the material culture within eating disorder settings. This theme critiqued eating disorder services, including idiosyncratic use of assessment methods, inaccessible service locations, and the limited number of beds available in specialist mental health services. The second theme, place, referred to the meaning given to social interactions created within space. Participants critiqued the privileging of non-Māori experiences, and how this makes a place and space of exclusion for Māori and their whānau in eating disorder services in New Zealand. Other barriers included shame and stigma, while enablers included family support and self-advocacy.

**Conclusion:**

More education is needed for those working in the space of primary health settings about the diversity of those with eating disorders to enable them to look beyond the stereotype of what an eating disorder looks like, and to take seriously the concerns of whaiora and whānau who present with disordered eating concerns. There is also a need for thorough assessment and early referral for eating disorder treatment to ensure the benefits of early intervention are enabled for Māori. Attention given to these findings will ensure a place for Māori in specialist eating disorder services in New Zealand.

**Graphical Abstract:**

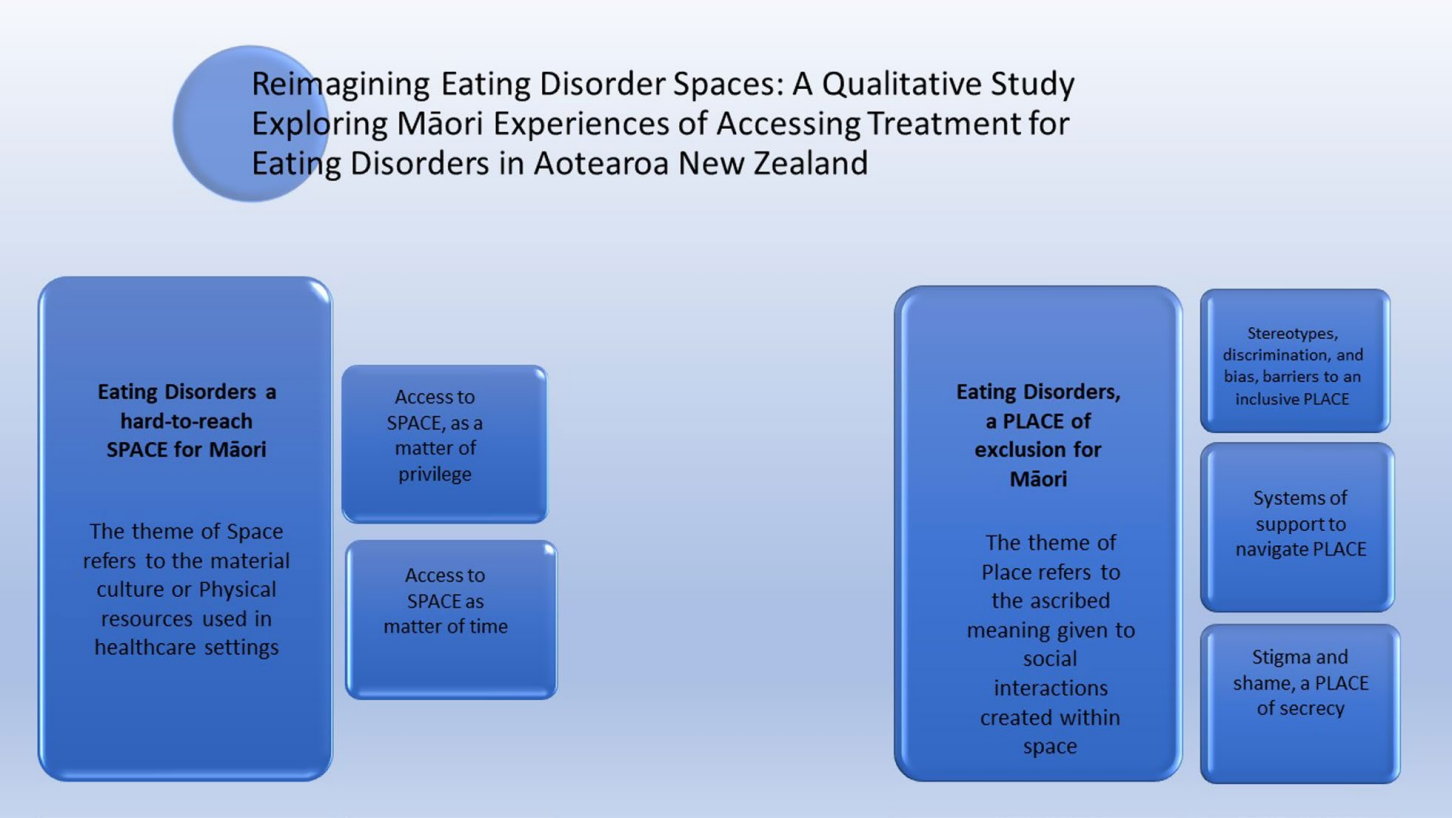

## Plain English summary

Eating disorders are at least as common in Māori (Indigenous people of New Zealand) when compared to their non-Māori counterparts, however, a recent study investigating specialist service use data identified lower-than-expected service use for Māori. This paper explores the lived experiences of Māori with eating disorders and their whānau (family/support network) to understand the barriers and enablers to accessing treatment. Participants in this study identified both systemic and social barriers to accessing treatment for eating disorders; Systemic barriers included the idiosyncratic use of assessment methods by health practitioners, and inaccessible service locations, including the number of available inpatient beds. While social barriers included the stereotype of what an eating disorder looks like, shame, stigma, and discrimination; support networks were described as both an enabler and barrier to accessing specialist treatment for eating disorders. The findings from this study suggest thorough assessment and early referrals are needed for Māori presenting with disordered eating concerns. Moreover, more education is needed for those working in primary healthcare settings about the diversity of eating disorders to ensure they move beyond the stereotype of what an eating disorder looks like.

## Introduction

Health, illness, and the body are conceptualized within the cultural context of a society. The values and belief systems of a society, including media portrayals, shape how health and illness present. Until relatively recently, eating disorders (EDs) were considered a culture-bound syndrome rooted in western societies [1, 2]. The international cultural phenomenon was said to be informed by western ideals of beauty, which included thinness for women [3]. The acronym SWAG (skinny, white, affluent girls) was coined and popularized in the press [4, 5]. Men, are said to strive for muscular or toned body types with low body fat [6, 7]. Overtime, this has resulted in a fat phobia discourse contributing towards the development and maintenance of over-evaluation of weight and shape that has been proposed as a driver of ED behavior [8]. As a result, national and international ED research has focused on, and privileged European/White experiences, including for those in settler colonial locales [9]. The antiquated approach to EDs has had a profound impact on both the presentation and assessment for EDs, including the marginalization of EDs experienced by diverse Indigenous and ethnic populations [10]. ED research undertaken over the last two decades now suggests that the prevalence of EDs is higher in minority ethnic groups when compared to their European/White counterparts [11–13]. High rates of disordered eating is also reported in ethnic minorities but they may not meet full criteria for EDs as defined in the fifth edition of the Diagnostic and statistical manual of mental disorders (DSM-5) [14], which may have an impact on access to treatment [11].

As a settler colonial nation, the New Zealand (NZ) health system has privileged the values and belief systems of its colonial heritage over and above those of Māori, the Indigenous people of NZ who account for 17.1% of the total population [15]. This privilege also extends to conceptualizations of mental health. The relationship between Māori and the British Crown (embodied by the NZ government) was formally outlined in Te Tiriti o Waitangi, the nation’s founding document. Historically colonization led Māori to be removed from their traditional lands, their culture to be marginalized from civic spaces and Te Reo Māori (Māori language) to be nearly lost. These historical acts, coupled with the ongoing process of colonization has resulted in non-Māori privilege in mental health status based on differential exposure to the social determinants of health [16]. The NZ healthcare system is characterized by universal health coverage and privatized healthcare. Universal health coverage is paid for by taxation, while primary healthcare services are subsidized for low-income earners and people with disabilities. Government funding covers the cost of inpatient and outpatient specialist mental health services, including ED services. Despite universal health coverage, and mandates to reform healthcare for Māori, health inequities including for physical and mental health outcomes remain pervasive [16].

To date, the most comprehensive population estimate of the prevalence of ED in NZ is based on Te Rau Hinengaro (the 2004 New Zealand Mental Health Survey). The report found that EDs were at least as common in Māori as in non-Māori for Anorexia Nervosa (AN) (Māori 0.7% non-Māori 0.6%), and Bulimia Nervosa (BN) (Māori 2.4%, non-Māori 1.3%), and for all EDs combined the Māori rate was almost double the rate of non-Māori (Māori 3.1%, non-Māori 1.7%) [17, 18]. However, a recent study investigating whole-of-nation specialist service use data identified lower than expected service use compared to the assessed crude prevalence of ED identified in the Te Rau Hinengaro report for Māori. This suggests a low rate of access to specialist ED services for Māori with ED, indicating the likely presence of systemic bias [19]. However, no published data about the barriers and enablers for Māori accessing treatment for EDs exists.

The purpose of this paper is to present an understanding of Māori experiences of ED, including whaiora (person in pursuit of wellness) and whānau (family/support network) perspectives on the enablers and barriers to accessing treatment for ED, to examine the systemic factors that contribute to the underrepresentation of Māori in ED services.

## Method

### Design

Kaupapa Māori research is an Indigenous methodology that privileges Māori world views and has been used with mental health research to position whaiora and their whānau as experts in the critique of mental health provision in NZ [20]. It provides a philosophical framework that challenges dominant non-Māori interpretations of research by critiquing the imposition of western authority and colonialism [21]. Kaupapa Māori research ensures that Māori maintain control over the development, implementation, and interpretation of the research and that there is a strong positioning of Māori cultural values and the use of tikanga (protocols) Māori [21]. It also requires researchers to present research findings within a strength-based frame at the individual/whānau and community level, in doing this, the research considers the broader systemic determinants of Māori health and wellbeing [22]. This positioning is a crucial factor that influences how kaupapa Māori researchers conduct all steps of the research process and, in particular, the analysis and presentation of the findings. However, the approach is not prescriptive in that it allows the use of various methods that can be chosen based on the study's objectives [20].

This study uses qualitative methods to explore how systemic factors impact Māori experiences of EDs. The findings presented here are part of a multi-phased mixed methods study: Tangata Kōmuramura: Māori experiences of EDs. The first phase identified a low rate of specialist ED service use among Māori in NZ using a national dataset [19]. To explore this finding, in-depth, detailed qualitative interviews with Māori were conducted. A key focus of these interviews was the participant's experiences of barriers and enablers to health service use.

#### Recruitment and participants

Due to the underrepresentation of Māori in specialist ED treatment in NZ, recruitment efforts beyond sampling in health services were required. For this reason, purposive sampling was not applied and a variety of other recruitment strategies were employed. This included media stories, social media and website advertising, email distribution through Māori community networks, and the circulation of hard copy posters and brochures to various locations, such as Māori organizations, primary care waiting rooms, counseling services, university campuses, libraries, and venues of ED-related recovery groups.

Participants were recruited if they: self-identified as Māori ethnicity, were aged 16 years or over, and had either a lifetime diagnosis of AN, BN, or Binge Eating Disorder (BED) or self-identified as having one of these conditions. The youngest person in this cohort was aged 16 and the eldest was 65 years of age. Potential participants who had not been previously diagnosed (n = 4) were screened using the MINI international neuropsychiatric interview for ED [23]. Active recruitment of whānau who had supported whaiora with an ED was also undertaken. In line with tikanga Māori, all participants were provided with the option to be interviewed with whānau, but all participants elected to be interviewed individually and without further support. A koha (gift) in the form of a supermarket voucher to the value of fifty dollars (New Zealand) was offered to each participant. Koha is an act of reciprocity that acknowledges a person's contribution to the study while recognizing the mana (authority, prestige, status) possessed by each Participant.

#### Data collection

In line with kaupapa Māori research, interviews followed the hui process [24]. The hui process is a tikanga Māori-informed method of engagement designed to enhance the clinician-patient relationship. This process involves mihimihi (introductions and engagement), whakawhanaugatanga (building from elements of Te Ao Māori at the beginning and throughout the engagement that promotes mutual sharing between the interviewer and interviewee as a means of establishing a relationship that promotes power-sharing and trust to support appropriate informed and engaged consent), the kaupapa (the topic being discussed within the bounds of this research project and interview), and poroaki (consolidation of key points raised in the interview, what the participant’s role in the research is going (e.g. receiving transcript or report at end of study), and the aspirations for the research (e.g. timelines, policy influence, publication of findings). The kaupapa was structured around three pre-determined topics based on the objectives of the study, which included: (1) self-perceived causes of the ED, (2) access to treatment, and (3) experience of treatment. While there were some pre-determined questions based on these topics, the interviews had the flexibility of following the participant's preferred narrative in keeping with kaupapa Māori research. The interviews were conducted face to face (n = 4), via video link (n = 6), or over the phone (n = 5) and ranged from 36 to 89 min, with communication format based on location and participant preference. They were audio-recorded and transcribed verbatim.

#### Data analysis

The transcriptions were analyzed by three researchers (MC, JM, SP) using NVivo [25]. At the time of gaining informed consent, participants were offered the opportunity to review their transcripts. The first stage of analysis involved reading and re-reading the transcripts to become familiar with the data. Deductive structural coding [26] was then conducted by MC as a means of organizing the data within the preformulated research objectives. The data was therefore organized into three sections: (1) self-perceived causes of the ED, (2) access to treatment, and (3) experience of treatment. For this paper, only data specific to participants' experiences of accessing treatment were analyzed.

Inductive thematic analysis was utilized and is frequently employed in kaupapa Māori research because of its ability to align with kaupapa Māori research's theoretical positioning. In addition, the flexibility of the method allows the researcher to consider the latent meaning of the participant's experiences, including how they inform the impact of macro-level structural determinants on Māori health [20, 27]. The first cycle of inductive coding was descriptive and remained close to the participants' meanings that described what whaiora and/or whānau perceived to be barriers or enablers to accessing ED services. The descriptive codes were clustered according to barriers and enablers to accessing treatment. Pattern coding was then conducted to collate codes into categories. The relationships between the categories were then considered across the data, where two clear themes were determined. During this coding process, the researchers consciously considered what the data suggested about systemic factors that impact Māori experiences of EDs. Coding and theme development were conducted by MC, and reviewed by SP and JM.

After identification of key themes around barriers and enablers of access to treatment, Low’s spatializing culture theory was used to provide a framework for further interpretation of the findings [28].

### Data availability

The data that support the findings of this study are available on request from the corresponding author, [MC]. The data are not publicly available due to contents that could compromise the privacy of research Participants.

### Ethics

The study received ethical approval from Health & Disability Ethics Committee.

(16/N.T.B.189 AM02). Detailed information sheets were provided to all participants and signed informed consent was obtained. All identifiable information was removed from transcripts.

## Results

Fifteen people participated, comprising of 13 people with lived experience of an ED (AN n = 8; BN n = 4; BED n = 1) and two whānau members who supported their relatives with AN. The majority of participants were female (n = 14). The analysis of whaiora and whānau experiences identified two themes that illustrate self-perceived enablers and barriers to accessing ED treatment for Māori. The first theme, *Space*, critiques assessment methods including the subjective nature informing the assessment method used, service location, and the limited number of beds available in specialist services for EDs. In the second theme, *Place*, participants critique the privileging of non-Māori experiences and the implication that this creates including a *place* of exclusion for Māori patients with EDs and their whānau in ED *spaces* in NZ.

### Eating disorders, a hard-to-reach *SPACE* for Māori

*Space* was reported as the material culture or the physical resources used in health care settings. This included service settings, locations of specialist ED services, service integration, and assessment methods for EDs. Specifically, the concepts of access to *space* being a matter of privilege, and a matter of time were identified as subthemes that will be extrapolated within this section.

*Access to space, as a matter of privilege* was identified as participants critiqued the tools used by health professionals to assess EDs that determine inclusion/exclusion criterion for access to publicly funded specialist ED services. Participants accounts of their assessment and referral practices indicated that these practices were dependent on the ED-health literacy of the health professional, which in many instances created barriers to accessing further ED treatment. Testing measures used to assess for EDs within this cohort ranged from no test, enquiry into daily food intake, weighing and body mass index, depression scale test, blood tests, or electrocardiogram. For most participants, assessment methods included the use of one of these assessment tools, but for others, no objective assessment measures were used:

“I remember quite plainly one GP (general practitioner) saying to me ‘look don’t worry, I don’t think there’s anything going on, I usually get a feeling up the back of my neck when it’s something like an eating disorder and it’s not that’” TK10.

Participants expressed frustration when GPs privileged their own knowledge over and above the voices of whaiora and/or their whānau experiences because it resulted in multiple GP visits over extended periods, and delayed access to timely care:

“[GP said] No, her BMI is normal she’s fine. I said ‘she’s not fine she’s lost 10% of her body weight in the last two months, she is crazy and she’s not eating very much, I’m sure she has Anorexia?’ …. It wasn’t until she was admitted at the end of October, because I had taken her back three or four times and when we finally insisted on getting a CAMHS [child and adult mental health service] referral” TK03.

*Access to space as a matter of time* was noted as participants described the factors that contributed to delayed access to timely treatment for their ED. Location of services, limited beds, and comorbidities were identified as key factors. Wait times for inpatient treatment ranged from 1 to 6 months. Those in rural locations described a lack of ED expertise available including for secondary health services. Participants noted an observable difference in the quality of care in rural settings when compared to health professionals within specialist ED services. However, specialist ED services were located in urban centers. Accessing specialist ED services resulted in the separation of whaiora and whānau during periods of treatment.

“Definitely more regional support [is needed] so like… not in the cities, there is quite good support, but in places like [rural location] there’s not really anything. I’m not really sure about now but throughout the whole time I was sick, there wasn’t anyone who treated eating disorders and the hospital were quite difficult around it, so more understanding from medical professionals as well, like more education around it” TK13.

There was an overwhelming consensus among participants that limited beds in specialist ED services was a major contributing factor to delayed access to care. Some participants identified coexisting disorders as a factor that contributed to delayed access to care. For example, one participant reported that during inpatient treatment in specialist services for bipolar disorder, they could not gain access to specialist ED services until perceived issues relating to bipolar were addressed. This example documents the need for service providers to consider integrated approaches to healthcare, including better connections between services rather than a sequential siloed treatment approach:

*“More flow between eating disorders and other services, more connection because from my point of view I feel that eating disorders is quite separate and it's hard to get them involved and waitlist times are horrible. If I’m struggling and I get referred back there it's like oh cool it's going to be a three month wait and I’m like well, by the time it gets to that three months I’m like nah – whatever” TK01.*

Participants described wait times as distressing experiences due to the observation of the deteriorating health of whānau.

### Eating disorders, a PLACE of exclusion for Māori

*Place* was described as the location in which health care was delivered and its ascribed meaning for the participants. This was created through social interactions, memories, feelings, emotions, imaginings, and the use of the physical location. Ascribed meaning included societal views embedded within *place*. The variables helped to create spatial transformation which gave the *place* attributes that were identified as: promoting stereotypes, discrimination, and bias that acted as barriers to the service being an inclusive place; systems of support; or stigma, shame, and a place of secrecy. Each of these attributes are presented as subthemes of *place*.

*Stereotypes, discrimination, and bias, barriers to an inclusive place* were well noted by Participants as attributes that contributed to experiences of bias and discrimination resulting in delayed access to care. Participants identified two social narratives that acted as barriers to accessing treatment. These included assumptions about what a typical ED looks like including whom EDs affect, as well as, factors contributing to the development of an ED. The latter considered societal-held assumptions about the value of food in Māori culture. Participants identified cultural stereotypes as not only additional barriers to access, but also barriers applied exclusively to Māori. Participants acknowledged the impact of EDs as a perceived ‘western-bound syndrome’, not only based on their own understanding of EDs, but also on how these biases are held within wider society including for health professionals.

*“I think I found it made me quite like a little bit annoyed because I felt like people had this idea that it affected European teenage girls and they had to be quite wealthy and like all those strange kinds of stereotypes. Like, even before I was ill, I thought the same thing” TK13.*

*“I don’t know whether, if they would have taken more action if I had looked different or if – and I gotten me help more immediately” TK09.*

Participants expressed frustration with the notion that Māori do not experience EDs because of the role of food within Māori culture. Participants noted that food is present in all Māori social gatherings although not exclusive to Māori culture.

*“I mean we had some jokes like I never heard of a Māori anorexic before. There’s food everywhere, but that’s in every culture” TK03.*

*“I had this feeling like I’d be judged more because I am Māori and Māori should – we like to eat lots and we shouldn’t have any issues around food” TK08.*

Participants' experiences tell us that the prioritized narrative within ED has focused heavily on European experiences. This resulted in internalized negative beliefs that rationalize or seek to confirm why EDs do not exist in Māori. The unintended consequence has resulted in some whaiora questioning affiliation to Māori identity.

*Systems of support to navigate place*, describes the multiple systems of support required to enable whaiora and/or whānau to successfully navigate health care settings for access to ED treatment. Participants identified systems of support as both enablers and barriers to accessing specialist ED services for ED treatment. The systems included family and friends, or health professionals within primary healthcare settings. Characteristics of positive interpersonal interaction with health professionals included empathy, and continuity of care. Positive characteristics of whānau support, including friends, are described as acting swiftly to Participant concerns by aiding or taking an active lead in navigating systems in *place.*

*“So, I reached out to my parents for some help. I have got a really good relationship with my parents. And let them know I had been struggling and then kind of the whole clinical process started so that was an interesting one, too, because the day after I told my parents that I had been purging all of my food, my Mum took me to the doctor” TK11.*

*“I think school was such a struggle so having someone if something was going wrong at school then I could just go to and I just really clicked with her and it was just nice to have someone to talk to because I didn’t have anyone, any professionals involved in my care at that time” TK01.*

When systems of support were insufficient, self and/or whānau advocacy was needed to overcome barriers to accessing treatment. Participants described this process as persistence. Persistence entailed ongoing consultations with GPs, demanding access to care, or learning to navigate the system that allowed for a bypass of gatekeepers to specialist treatment for EDs.

*“I’m angry, but I also want to educate people so that no other family have to go through what we’ve been through to get treatment. I mean for God’s sake I work in the health system and we got screwed over at every corner. I knew what I was doing, I knew who to ask what and I still you know got bamboozled” TK03.*

Participants acknowledged the pivotal role of health professionals and whānau in gaining access to treatment for EDs. However, when systems of support are not available, self and/or whānau advocacy are required to navigate the systems in *place*.

*Stigma and shame, a place of secrecy*, in this subtheme Participants described the impact of stigma on their ability to share ED behaviors or concerns. Stigma was stated to result in shame, which supported secret disordered eating behaviors. Stigma was related to a perceived hierarchy amongst EDs. For example, AN was often perceived as an ED to fear notably as a result of one’s fragile appearance. Participants agreed that thinness was perceived to be a marker of illness severity. The messaging was experienced within the Participants’ social environment and through interactions with health professionals within primary healthcare settings. As a result, those with BN or BED claim their illnesses were less valued.

*“I just think a lot of people were scared of because when people say anorexia, you have these images in your head of someone so frail and you don’t want to hurt them. There’s like a scare around it. And especially being Māori” TK07.*

*“…that’s how long it has taken me to really, that’s how much shame I had attached to that. And even though I hadn’t actually like purged since I was maybe 24. I still feel so embarrassed that I had had this disorder and that I felt like I had to do that” TK08.*

Enablers to accessing treatment included health promotion and health literacy. Some Participants expressed empowerment through TV personalities sharing their lived experiences of mental health issues including EDs. Explanatory frameworks that included exposure to childhood adversity as contributing factors to EDs development was viewed by participants as reflective of their own experiences. The lived experiences of diverse populations assisted in creating narratives that whaiora could relate to, encouraging acts of self-disclosure amongst family and friends.

*“No-one wanted anything to do with it until I posted and was really open about it. And it was weird because I saw a Mike King [NZ comedian, advocate for mental health awareness] thing and it was something about him saying that you need to be vulnerable and something like that, and I was like, you know what, yeah I am sick of being embarrassed of this. And that kind of opened up the gates for me to start recovering, I guess. Because there was nowhere to hide anymore” TK07.*

## Discussion

This study drew on whaiora and/or whānau experiences of an ED concerning barriers and enablers of treatment for ED for Māori. The thematic analysis results were framed within overarching themes—‘*space*’, and ‘*place*’. Low’s ‘spatializing culture’ framework was used to interpret these findings [28]. Theme one, “EDs, a hard-to-reach *space* for Māori” is informed by the material culture within primary health settings for EDs. Participants critiqued the adequacy of, or idiosyncratic ED assessment methods used, reflecting health professional ED-health literacy; service location affected ease of access, and insufficient availability of beds within inpatient settings. Participants considered these sub-themes as barriers to accessing treatment for EDs. Theme two, “EDs, a *place* of exclusion for Māori”, is informed by the ascribed meaning given to space which is created through social interaction, memories, or imaginings. Participants described stereotypes, discrimination, bias, stigma, and shame as barriers to inclusion, while enablers were described as good support systems, health promotion, and ED-health literacy.

This study provides rich qualitative data that supplements our previous studies' findings that identified reduced access to treatment for Māori with EDs [19, 29]. The findings suggest both unconscious, interpersonal, internalized, and systemic bias are likely contributing factors to the low rates of healthcare access. These results suggest that part of this bias may be related to continuing use of diagnostic frameworks for EDs derived largely from samples of EDs in white urbanized international populations [4]. We found here, that participants who had an ED other than AN, described their ED as being not recognized when they sought help. Similarly, in Australia among Aboriginal and Torres Strait Islander peoples, higher rates of disordered eating have been identified but they are more likely to receive a diagnosis of other specified feeding eating disorder or unspecified eating disorder than other ethnicities [30]. These types of diagnostic differences may create barriers for people who do not fit the privileged diagnostic framework, given diagnostic thresholds are often used to triage treatment.

This study identified difficulties in whaiora or whānau having the presence and/or seriousness of the ED recognized by GPs, resulting in delayed treatment. There is strong evidence that early intervention achieves the best outcomes for EDs [30, 31]. However long periods of time from the onset of an ED to accessing help are common, with around half taking up to a year to seek help [31, 32]. In NZ specifically, evidence suggests delays in accessing care are particularly long for those with BN and BED [33]. In an environment of long wait times, failure to adequately screen for an ED and/or delayed referral means that the ED is likely to become entrenched, so reassurance or a wait-and-see approach is unhelpful. This poses a significant barrier for whaiora and whānau in accessing treatment, thereby perpetuating disparities in health and equity outcomes for Māori with EDs [34]. Referring to specialist care early is essential [35]. Although AN is less likely to be missed due to visual evidence of underweight status, there is evidence that BN and BED and subthreshold but clinically significant variants in other specified feeding or eating disorders are likely to be missed [36]. This may be particularly so in those with EDs and higher body weight. It was recognized in recent guidelines that those with ED and higher body weight, are more likely to be offered weight loss advice but not screened for an ED [36], which reinforces stigma. Untreated BED is associated with increasing weight over time so early treatment has important long-term health benefits for individuals [37]. Routine screening for BED is important to ensure BN and BED are adequately assessed and referred for treatment.

In NZ, there are a limited number of inpatient beds with only four publicly funded specialist services for EDs which are located in New Zealand’s three largest urban centers. One in four Māori live in rural areas [15], and our findings indicate living in a rural area could be an important barrier to access for Māori in need of ED treatment.

The strengths of this study include the use of kaupapa Māori methodology to validate the voices of Māori through centering the analysis from a Māori worldview, as well as, critiquing the impact of colonial structures on health service delivery and outcomes. Due to current inequity and a lower rate of Māori accessing ED services, the recruitment into this study was complicated and required multiple community networks and advertising, which resulted in a small number of participants and limited coverage across AN, BN, and BED diagnoses. Participants were provided an opportunity to review their interview transcript, however, no Participants opted to review their transcript. Self-selection into the study means that it is difficult to determine how these participant voices reflect the broader Māori experience of ED accessing treatment.

## Conclusion

The findings from this study have implications for clinical practice in NZ. More education is needed for those working in the space of primary health settings about the diversity of those with EDs to enable them to look beyond the stereotype of what an ED looks like, and to take seriously the concerns of whaiora and whānau who present with ED’s. There is also the need for thorough assessment and early referral for ED treatment to ensure the benefits of early intervention are enabled for Māori with EDs. Attention given to these findings will ensure a *place* for Māori in specialist ED services in NZ.

## Data Availability

The data that support the findings of this study are available on request from the corresponding author, [MC]. The data are not publicly available due to contents that could compromise the privacy of research participants.

## Appendix

Hui: Ceremonial gathering.

Kaupapa Māori: Kaupapa Māori relates to the knowledge, attitudes and values that are inherently Māori

Koha: Gift

Māori: Indigenous people of New Zealand.

Mana: Authority, prestige, status.

Mihimihi: Speech or greeting.

Poroaki: Farewell, closing.

Te Ao Māori: The Māori world-view.

Te Reo Māori: The Māori language.

Te Tiriti o Waitangi: Treaty of Waitangi.

Tikanga: Customary practices or behaviors.

Whaiora: Person in pursuit of wellness.

Whakawhangaungatanga: The process of establishing relationships.

Whānau: Family/support network.
